# Effects and Complications of Subcutaneous Implantable Cardioverter-Defibrillator in the Prevention of Sudden Cardiac Death: A Narrative Review

**DOI:** 10.7759/cureus.30170

**Published:** 2022-10-11

**Authors:** Prasad A Pagore, Shilpa A Gaidhane

**Affiliations:** 1 Medicine, Jawaharlal Nehru Medical College, Datta Meghe Institute of Medical Sciences, Wardha, IND; 2 School of Epidemiology and Public Health, Jawaharlal Nehru Medical College, Datta Meghe Institute of Medical Sciences, Wardha, IND

**Keywords:** subcutaneous implantable cardioverter-defibrillator, effects and complications of defibrillator, indications of defibrillator, sudden cardiac death (scd), ventricular arrhythmias

## Abstract

An implantable cardioverter-defibrillator (ICD) is one of those devices that is a big boom for the prevention of sudden deaths due to heart failure. This particular device has been in use for just a couple of years, but its impact in the domain has brought about a considerable change in the way a specific issue of the cardiovascular system is tackled. Although subcutaneous or hypodermic implantable cardioverter-defibrillator (S-ICD) is considered to be a better alternative as far as transvenous implantable cardioverter-defibrillator (TV-ICD) is concerned, the former, being a newer introduction in the market, needs to be assessed in depth to clearly understand its effects and complications. Various types of research have been conducted on the efficacy of this device, and in most of the studies, the supremacy of this device is clearly evident when compared with other devices that are used for the same purpose. Better innovations in subcutaneous or hypodermic implantable cardioverter-defibrillators would enable them to be manufactured in a more efficient and cost-effective way so that a huge lot of people are benefited from this device. This review article is a whole peep into the various studies done in this domain, thereby providing adequate scientific insights about subcutaneous or hypodermic implantable cardioverter-defibrillators in a very simple and comprehensive manner.

## Introduction and background

The Food and Drug Administration (FDA) has recently accepted a widely proven and patient-friendly device, the subcutaneous implantable cardioverter-defibrillator (S-ICD). It is a fully extrathoracic device for preventing the episode of sudden cardiac death (SCD), one of the leading causes of death in developing countries in cardiac patients [1]. The transvenous implantable cardioverter-defibrillator (TV-ICD) was previously used. The transvenous implantable cardioverter-defibrillator is being dominantly substituted by the S-ICD because the vasculature of the heart is not evenly touched during its procedure, so it causes minimum damage to the heart [2]. It is an amazing technique because similar to other treatment modalities, the lead is placed or implanted in the right ventricle, which obviously needs surgical intervention. This is dramatically reduced in this newer technique and reduces the incidence of complications. S-ICD is similar to the transvenous implantable cardioverter-defibrillators, excluding patient populations who need excitation just because of conductivity disorders or ventricular tachycardias that can effectively be treated by anti-tachycardia pacing [3]. Patient populations who have an indication of an implantable cardioverter-defibrillator (ICD) mostly have a serious cardiac disorder with hypertrophic left ventricular dysfunction, along with many other vascular diseases. Hence, a basic knowledge of the function of the device is essential for medical professionals as device functionality and proper placement should be known to identify the malfunction and malposition of the device, respectively. These patients will mostly visit a local practitioner to be checked in the emergency room, in the primary healthcare unit, or in the hospital by a medical professional other than a cardiac disease specialist [4].

## Review

Patient selection

The subcutaneous or hypodermic implantable cardioverter-defibrillator (S-ICD) was invented and is applicable for preventing sudden cardiac death among the patient population. Patients implanted with this device were apparently in minor age groups and have advanced heart diseases [5]. Patients who require the implant are those with ischemic heart disease, a QRS complex width greater than or equal to 150 ms, and a left bundle branch block (LBBB). Patient factors in which an ICD implant is not required are limited life expectancy, myocarditis for less than six months, and QRS width of less than 120 ms [6].

Subcutaneous or hypodermic implantable cardioverter-defibrillator system

The subcutaneous or hypodermic implantable cardioverter-defibrillator (S-ICD) has a subcutaneously placed titanium-coated pulse producer and is subcutaneously implanted. The lead is made up of a proximal and a distal differentiating conductor part apart from each other with an approximately 3-inch shock coil. The subcutaneous or hypodermic implantable cardioverter-defibrillator (S-ICD) does not cure bradycardia. Rather, it can generate or produce pacing, which is transthoracic shock, for up to 30 seconds. The machine consists of configurable areas of anti-bradycardia detection, which are mostly in the area of ventricles that occur during the fibrillation of ventricles. Heart rate is a parameter to detect whether we should deliver direct current (DC) shock or not in the ventricular fibrillation (VF) area [5].

Sudden cardiac death

Recently, sudden cardiac death (SCD) is becoming a significant problem in premature age groups [7]. According to the current statistics and data, between 300,000 and 400,000 people die in the United States annually, and about 350,000 deaths occur in the population of Europe [7]. It is a leading cause of death in developing countries such as India. Sudden cardiac death is a big trauma to the family, and along with the family, it also harms the country’s health system [8]. Ventricular arrhythmias and sudden cardiac death are significantly associated with structural heart disease. Research findings have also shown that the likelihood of sudden cardiac death increases during the early morning when sympathetic nervous system activity and premature repolarization rise and cause calcium overload [9]. The incidence or rate of occurrence of sudden cardiac death increases in heavy runners or marathon runners [10]. Severe acute respiratory syndrome coronavirus 2 (SARS-CoV-2) may lead to myocarditis, and this is an important cardiac manifestation that is linked with sudden cardiac death (SCD) [11].

Pathophysiology of sudden cardiac death

**Figure 1:**
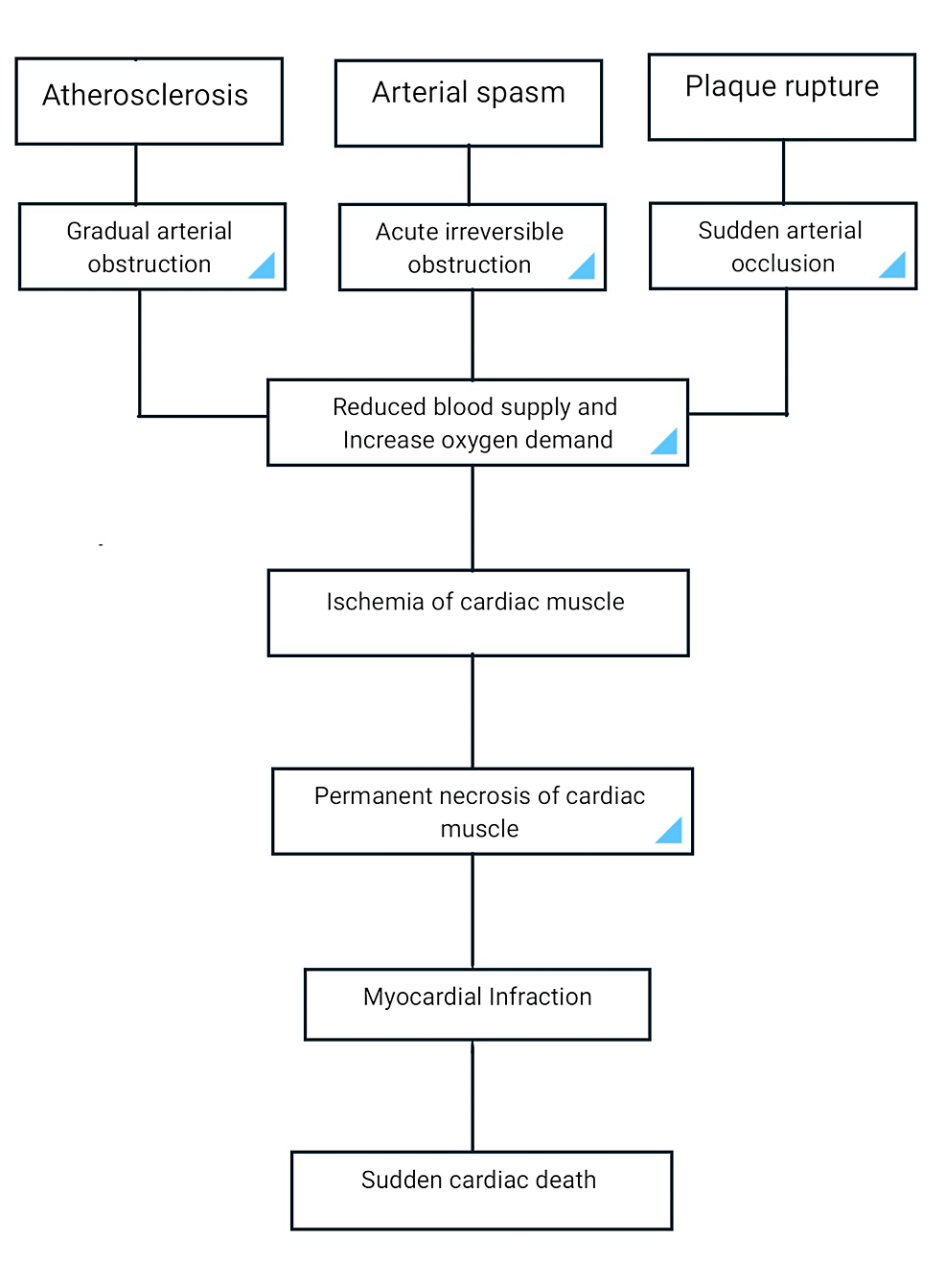
Flowchart showing the pathophysiology of sudden cardiac death [12]

The episode of sudden cardiac death increased after myocardial infarction (MI) in the first few months because of life-threatening tachyarrhythmias, recurrent infarction, or myocardium tear. Fibrillation of the ventricle and tachycardia were considered to be the main causative factor of sudden cardiac death. However, according to studies, loss of pulse (pulseless) electrical activity (PEA) and lack of systole or contraction are more common causes [12]. Figure 1 shows a flowchart that describes the pathophysiology of sudden cardiac death.

Efficacy of the device (S-ICD)

The decrease in mortality rate due to SCD is the sole determinant of an ICD’s potency, but knowing these statistics hardly requires long follow-up studies and research projects, which are currently unavailable for a completely newly launched device such as S-ICD. Significant cohort studies that have multicentric participants with S-ICDs are presently being researched, and the outcomes will offer preliminary information on the device’s actual effectiveness. Defibrillation threshold testing (DFT) is performed when a device is implanted to determine shock effectiveness in the event of ventricular tachyarrhythmia. If one or more than one shock is required to treat the arrhythmia, the S-ICD can very efficiently perform with each subsequent shock, which all has a power generation of 80 J [13]. The implantable subcutaneous cardioverter-defibrillator system has the power of delivering post-shock pacing in a mode of demanding at 50 ppm for up to 30 seconds after shock [14]. All of the main benefits of the subcutaneous defibrillator system are subsequently reduced. Similar with TV-ICD, it has lead issues, which are mainly the failure of lead and occlusion in veins. Skin deterioration and inflammation due to device removal contributed to 4.1% of all S-ICD implantation complications, making them the most infrequent ones. Of the complaints, 1.1% were other procedural problems. Four in 60 patients experienced implantation-related problems or misplacement of the parasternal S-ICD lead or pulse generator, which may require total reimplantation. Despite the limited follow-up time, these findings indicate how safe the device implantation is with intermuscular groove formation.

Only one (1.7%) patient in the latest research experienced an adequate shock for VF during follow-up. The following are some potential explanations for the study’s lower incidence of significant shock. In comparison to the recent S-ICD populations and studies, the median follow-up length is shorter, at 275 days, another factor connected to the underlying heart condition. In the research, S-ICD was used to prevent sudden death secondary to prior MI in almost 60% of eligible patients. Only one patient experienced an adequate shock during follow-up. Therefore, the S-ICD system’s defibrillation efficacy was confirmed by the success rate of defibrillation tests [15]. Implantation of the S-ICD intramuscularly could be long-lasting, is safe concerning infection, and is the best alternative to the standard subcutaneous placement [16].

In one study in which the statistical data of 803 people was included, of which 23% were female and 52% had ischemic heart disease, the mean ejection fraction (EF) came out to be 23.6%. When follow-up was taken for 41.1 months, the result was that 25.7% survived by delivering the appropriate shock and 14.1%, unfortunately, died just because they did not receive any shock when needed. Some patients received the shock but then also died immediately because of inappropriate shock [17,18].

According to some authors, as age advances, the efficacy of the implantable cardioverter-defibrillator is effectively reduced considering geriatric syndrome [19]. People with a high degree of heart failure with manifestations that do not respond to appropriate standard treatment and those whose heart transplantation does not work should never have an implantable cardioverter-defibrillator intervention, although some of these patients might still be offered the option of receiving cardiac recombination therapy with ICD backup. The majority of these patients eventually die from progressive catastrophic failure. However, the research of Sweeney et al. indicated a modest increase in longevity for people with advanced heart problems after ICD implantation. Additionally, it is not recommended for individuals with medically drug-resistant ventricular tachycardia or irregular heartbeat to utilize an ICD. Transplanting an implantable cardioverter-defibrillator has not yet been evidenced to be effective for populations with significantly reduced systolic function, coronary vessel disease, and no symptoms of prolonged or non-sustained ventricular tachycardia, which are scheduled for coronary revascularization [20].

Complications

Any disadvantageous clinical occurrence integrated into ICD implant placement and function was recognized as a complication. The prevalence rate of inappropriate shocks, which are defined as an ICD shock for any condition other than ventricular arrhythmias or sustained ventricular tachycardia, is not regarded as an ICD-related problem, but rather as a distinct unfavorable event. Complications were divided into major and minor [21]. Over a median follow-up of 28.7 months (interquartile range: 25.2-33.7 months), 230 issues have been reported in 195 (13.5%) patients at a rate of 6.7 per 100 persons. Patients experienced their first issue 14 days (interquartile range: 1-96 days) after the ICD was installed [20]. Early ICD complications can also have some differential diagnoses, such as ventricular arrhythmia, myocardial infarction, cardiomyopathy, spontaneous coronary dissection, and pulmonary embolism [21].

At one of the 1,473 healthcare facilities that took part in an ICD registry, 4,375 specialists carried out 510,835 ICD surgeries for 498,379 patients between April 2006 and March 2010. The survey population is composed of 4,011 doctors who carried out 356,515 initial ICD implantations at one of the 1,463 hospitals after excluding 8,258 patient populations who had epicardial lead implanted, 133,363 patients who had formerly undergone ICD implantation, and 243 patients whose ICD implantation was carried out by a doctor who could not be identified [22,23]. According to available data, problems associated with ICDs were more common in complex devices than in single-lead devices [24,25]. In this extensive registry of ICDs implanted for primary prevention, DC-ICDs had greater rates of implant problems and generator replacements, but both groups’ rates of survival and inappropriate shocks were comparable [26]. Infection is a well-known risk factor for transvenous (TV) cardiovascular implanted electronic device (CIED) and a complication of CIED installation [27,28]. In four significant cohort studies that included patients from numerous nations in the early years of S-ICD availability, the rates of S-ICD infection were computed. During follow-up periods ranging from 30 days to 6.1 years following S-ICD implantation, infection rates varied significantly from 1.25% at six months to 6.8% at 6.1 years [29].

Management

Early Management (Less Than 30 Days Post-surgery)

In cases where S-ICD infection is not immediately visible, an antimicrobial therapy, usually cephalexin, is used as an initial course of therapy for superficial skin and soft tissue infection over about 7-10 days to see whether the local axillary dysfunction change for better or disappear. Trimethoprim/sulfamethoxazole is a strategy for treating those with a history of IgE-mediated penicillin or cephalosporin allergy. Clindamycin is an alternative treatment option for people who have a penicillin allergy, but it is not advised as it leads to an increased risk of *Clostridioides *chronic infection. To determine the patient’s history of beta-lactam allergy, an anaphylaxis consultation should be obtained if one has not already been. This is crucial since antibiotic prophylaxis will be advised for any subsequent CIED implantations, and antibiotic therapy may be beneficial in the long run. The rarity of systemic infection, which is characterized by increased morbidity and mortality and complicates S-ICD implantation, makes a trial of evidence-based oral antibiotic therapy reasonable as a first step in the medical therapy of cases that are not eventually diagnosed as definite S-ICD infection or as another diagnosis in the differential [26].

Late Management (More Than 30 Days Post-surgery)

Because S-ICD has only recently been used rarely, a profile of the frequency of factors that cause implant system changes that are indicative of delayed S-ICD infections is not yet available. Additionally, the initial evaluation of changes may be carried out by local practitioners instead of doctors skilled in the treatment of CIED infections, which might lead to deviations from the most effective management techniques. Complete device removal may be necessary for situations where implant site alterations are severe and getting worse. A tailored strategy that combines imaging procedures and multispecialty consulting is required in indolent instances. Imaging investigations should be acquired in these circumstances. Empiric antibiotic medication must be avoided since it can reduce the specificity of imaging [27].

Total surgical removal of the device

S-ICD surgical withdrawal normally does not require any specific tools or particular surgical skills. One case report describes an S-ICD electrode that needed to be removed using a mechanical sheath because of fibrosis around the coil and distal tip [30]. In another study, 32 patients underwent S-ICD lead extraction procedures. The median time after S-ICD lead insertion was 9.3 months (5.4-17.5 months). In 96.9% of patients, primary complete removal of the material was accomplished, and there was only one procedural failure (3.1%). Nine (28.1%) patients required a mechanical sheath to remove lead adhesions around the coil, while three (9.4%) patients required an additional incision. Simple traction of the S-ICD lead was successful in 19 (59.4%) patients. There were no difficulties with the surgery. Patients who had successful simple traction extraction had more recent implants (7.1 months versus 16.5 months, p = 0.04) and had less prior history of sternotomy (two months (10.5%) versus five months (38.5%), p = 0.09) [31]. The availability of numerous extraction tools has made transvenous lead extraction (TLE) techniques, which have been detailed using a step-by-step methodology, easier to perform and safer [32], although there is a non-negligible risk of both mild and significant problems with TLE [32]. After 50 years of practice, a step-by-step method is utilized for TLE extraction, first with straightforward traction, then moving on to the use of non-powered instruments, and finally moving on to powered equipment or the femoral approach [33]. Complications have been noted, and as S-ICD therapy has gained more expertise, it may occasionally be necessary to relocate or remove the subcutaneous lead [34]. For leads implanted within the last year, simple manual traction is frequently effective; however, for leads placed for an extended period, additional extraction equipment is frequently required [35].

## Conclusions

Subcutaneous implantable cardioverter-defibrillators play the main role in preventing sudden cardiac death. The advancement in this field has led to the prevention of a greater number of deaths. This method should be preferred more than the other existing modalities. This device has minimal complications because it minimally interacts with heart vasculature. Sometimes, one of the leads may get infected or be displaced from its ideal place. Such conditions have minimal chances of occurring and can be managed with small surgical procedures. S-ICD tracks the patient’s electrocardiogram (ECG) and delivers shock whenever required without any disturbance to the patient. As it is a costly device and not affordable for all, all we need is the effectiveness of S-ICD and an affordable device so that everyone can financially accept it. As of now, it is an excellent device and prevents the incidence of sudden cardiac death very effectively.
